# Four-factor risk score for the prediction of interstitial lung disease in rheumatoid arthritis

**DOI:** 10.1007/s00296-023-05313-6

**Published:** 2023-04-18

**Authors:** Gouri Mani Koduri, Anna Podlasek, Shyanthi Pattapola, Jufen Zhang, Deena Laila, Anupama Nandagudi, Shirish Dubey, Clive Kelly

**Affiliations:** 1 Rheumatology Department, Southend University Hospital, Prittlewell Chase, Westcliff on Sea, Southend-on-Sea, UK; 2 Rheumatology Department, Basildon and Thurrock University Hospital, Basildon, UK; 3 Nottingham Biomedical Research Centre, University of Nottingham, Nottingham, UK; 4 Department of Rheumatology, Oxford University Hospitals NHS FT, Windmill Road, Oxford, OX3 7LD UK; 5 Rheumatology Department, James Cook University Hospital, Middlesbrough, UK; 6 School of Medicine, Anglia Ruskin University, Cambridge, UK; 7 Nuffield Department of Orthopaedics, Rheumatology and Musculoskeletal Sciences, University of Oxford, Windmill Road, Oxford, OX3 7HE UK; 8 Tayside Innovation MedTech Ecosystem, University of Dundee, Dundee, Scotland

**Keywords:** Rheumatoid arthritis, Extra-articular manifestation, Interstitial lung disease, Risk prediction, Probability score

## Abstract

**Objective:**

Interstitial lung disease (ILD) is one of the commonest systemic complications in patients with rheumatoid arthritis (RA) and carries a significant morbidity and mortality burden. We aimed to identify key variables to risk-stratify RA patients in order to identify those at increased risk of developing ILD. We propose a probability score based on the identification of these variables.

**Methods:**

A retrospective, multicentre study using clinical data collected between 2010 and 2020, across 20 centres.

**Results:**

A total of 430 RA (210 with ILD confirmed on high-resolution computed tomography (HRCT)) patients were evaluated. We explored several independent variables for the risk of developing ILD in RA and found that the key significant variables were smoking (past or present), older age and positive rheumatoid factor/anti-cyclic citrullinated peptide. Multivariate logistic regression models were used to form a scoring system for categorising patients into high and low risk on a scale of 0–9 points and a cut-off score of 5, based on the area under the receiver operating characteristic curve of 0.76 (CI 95% 0.71–0.82). This yielded a sensitivity of 86% and a specificity of 58%. High-risk patients should be considered for investigation with HRCT and monitored closely.

**Conclusion:**

We have proposed a new model for identifying RA patients at risk of developing ILD. This approach identified four simple clinical variables: age, anti-cyclic citrullinated peptide antibodies, Rheumatoid factor and smoking, which allowed development of a predictive scoring system for the presence of ILD in patients with RA.

**Supplementary Information:**

The online version contains supplementary material available at 10.1007/s00296-023-05313-6.

## Introduction

Rheumatoid arthritis (RA) is a systemic inflammatory and autoimmune disorder that affects up to 1% of the general adult population worldwide. Pulmonary involvement is a common extra-articular manifestation of RA [1]. Respiratory manifestations include pleuritis, pleural effusions, bronchiectasis, nodules and Interstitial Lung Disease (ILD), of which ILD has the most impact on morbidity and mortality. ILD is clinically evident in approximately 10% of the RA population [2–6], accounting for 13% of the excess mortality [2, 4, 6–9] compared to the general population. Additionally, one-third demonstrate subclinical ILD on high-resolution computed tomography (HRCT) chest scans [2, 10–12]. ILD can occur at any point in the natural history of RA. Early diagnosis can be challenging, given that clinical manifestations may be delayed until the lung disease is well established. Radiographic and physiological changes as evidence on lung function may precede symptoms by years, and significant numbers of RA-ILD patients may be asymptomatic [13, 14]; however, once clinically apparent, ILD is associated with significant mortality [7] and functional impairment [2].

A model to predict the risk of developing ILD in individuals with RA patients could prove invaluable. Accurate risk prediction is central to guiding appropriate assessments, enabling more accurate patient prognosis and personalisation of care. It also informs the introduction of appropriate therapeutic intervention. To date, despite the high risk of developing ILD, a systematic approach to this problem has not been routinely adopted in clinical practice. A simple probability score or risk calculator, analogous to Q Risk for cardiovascular disease, that could be used during routine clinical assessment to identify high-risk patients would facilitate diagnosis and inform subsequent clinical care. We know from previous studies that RA-ILD is known to occur in older individuals with a four-fold relative risk for individuals above 65 years old [9, 15]. There is typically a male predominance [16–18], and smoking has been an independent risk factor in most studies, which may be dose-related (> 10 pack-years) [5, 10, 19, 20]. Disease-specific factors, such as the presence of erosive joint disease and rheumatoid nodules, have also been associated with ILD development [21, 22]. A few studies demonstrated associations with high RA disease activity [3, 20, 23, 24], functional impairment [14] and disease duration, onset of ILD being more frequent during the first 5–10 years of RA progression [19, 25]. Laboratory markers such as high titres of rheumatoid factor (> 100 IU/mL) significantly increased the risk of RA-ILD [3, 15], and this has also been reported for anti-cyclic citrullinated peptide (anti-CCP) antibodies [15, 17, 19, 26–28].

In addition to RF and anti-CCP antibodies, the usefulness of other potential biomarkers predictive of the onset of ILD is being investigated, most notably antibodies directed against carbamylated proteins (anti-CarP), serum levels of extracellular matrix metallopro-teinase 7 (MMP-7) , interferon gamma-inducible protein-10 (IP-10) or CXCL10, interleukin-18 and 90- and 70-KDa heat shock proteins (HSP90/70) [29–32]. None of these are yet available in clinical practice, nor have they yet been shown to have greater predictive value. Likewise, some genetic biomarkers have been identified, including mutations in the MUC5B gene [33, 34].

## Objective

We propose a probability score intended to help risk-stratify RA patients into those with a high versus a low probability of associated ILD. This is based on long-term experience of running an ILD MDT service and supported by the relevant literature as described in the introduction.

Established risk factors already exist in the literature, so this proof-of-concept study aimed to risk-stratify these individual factors weighing them to produce a probability scoring system.

## Methods

We performed a retrospective, multicentre study using data collected from the clinical notes of 430 consecutive patients with RA who fulfilled ACR/EULAR 2010 criteria [35]. Patients that had undergone HRCT from Southend, Coventry, Basildon hospitals and 17 other Hospitals contributing to the BRILL database between 2010 and 2020 were included in the study. Clinical suspicion of ILD was based on patient reported symptoms and signs, including dry cough, shortness of breath and the presence of any inspiratory crackles, and hence all these patients subsequently had further imaging with HRCT.

HRCT is the gold standard for diagnosing ILD and Chest radiograph (CXR) is the least sensitive imaging, but there are no specific guidelines for screening ILD in Rheumatoid patients. Controls with HRCT but other lung abnormalities such as Bronchiectasis or Emphysema were excluded from the analysis to minimise bias. CT follow-up plays an important role in detecting complications, such as pulmonary hypertension, pulmonary embolism, neoplasms and coronary artery disease, all of which can have an impact on survival. Many issues have yet to be resolved, including interobserver variation in the interpretation of scans and the optimum time interval for CT follow-up. Quantitative imaging with machine learning, is a new and promising approach, allowing quantification of patterns and the extent of the disease, which might eliminate the inter-observer bias.

This is a multicentre retrospective study. The cases were selected over a period of 10 years; hence, the centres have not used any specific criteria for defining ILD. ILD HRCT definition criteria have evolved over time. Thus, this presented a difficulty for HRCT standardization across our patient cohort spanning a 10-year period.

### Cases

ILD diagnosis was based on HRCT at any point during their disease duration. An experienced radiologist reported the scans describing the disease pattern and the extent of ILD. If there was any uncertainty, the scans were discussed again in an ILD MDT, and a consensus achieved.

Usual Interstitial Pneumonia (UIP) was defined as subpleural reticular opacities, associated with honeycombing and traction bronchiectasis with peripheral and lower lobe predominance [36]. Non-specific interstitial pneumonitis (NSIP) was defined as ground glass opacities either symmetrical, asymmetrical, diffusely distributed, or basal predominance. Fibrotic NSIP was associated with reticular opacities and traction bronchiectasis [37].

### Controls

Those with no ILD on HRCT were included in the control group.

We then collected data on the demographics: sex, age at RA onset, Disease activity score (DAS28) at RA onset, C-reactive protein (CRP) or Erythrocyte sedimentation rate (ESR) based on RA onset. Normal and abnormal values for rheumatoid factor (RF) were determined by local laboratory standards: rheumatoid factor titre category (positive > 42, weak positive 15–42, negative≤14). The cut-off values for anti-cyclic citrullinated peptide (anti-CCP) antibodies were based on American college of rheumatology criteria definition. Negative: less than or equal to the upper limit of normal (ULN); weak positive > ULN; positive > 3 × ULN. Anti-CCP titres were categorised as (positive > 21, weak positive 8–21 or negative≤7).

Data on medications (disease-modifying antirheumatic drugs (DMARD) or biologics), presence of erosions (ever) and smoking history (current/former/non-smoker/number of pack-years) were collected at the time of inclusion. The study commenced in January 2021 and completed in March 2022. The outcome variable was prediction of the presence of ILD in patients with RA. As this was a retrospective study, adequate data were unavailable on disease duration, time to develop ILD, Health Assessment Questionnaire (HAQ) scores, body mass index and occupation. To minimise any resulting bias, controls were unselected.

### Statistical analyses

Continuous variables were reported as mean and standard deviation (SD) or median and interquartile range (IQR). Categorical variables were reported as number and percentage. Comparisons between groups were performed using a *t*-test, Mann–Whitney test, and Chi-square, as appropriate.

Univariate and multivariate logistic regressions were performed, with the co-existence of RA and ILD as the dependent variable. Other variables (sex, age category, smoking status, RF titre, anti-CCP titre and DAS 28) were analysed as independent variables. Continuous variables were categorised according to cut-off points defined referring to previous studies [15–17]. Hosmer–Lemeshow tests were used to assess the goodness-of-fit of the logistic regression models. Statistical analyses were performed using R and Stata 17 statistical software, and a two-sided *P* < 0.05 was considered significant in all analyses.

### Patient and public involvement

Patients and public were not involved in the design and methodology of this study.

### Ethical approval

Ethical approval was obtained for Mid and South Essex hospitals Trusts [18075], Coventry Hospital Trust [GF 0265], and approval for the BRILL database has been previously described [38] This study is limited to a retrospective use of information previously collected during normal clinical care with no patient identifier recorded in the database for analysis. The study, therefore, did not require Research Ethics Committee review or formal patient consent.

## Results

Out of a total of 430 patients, 210 (48.8%) patients had RA-ILD as defined by HRCT, and 220 (51.2%) had no ILD.

Other lung pathologies observed in the control group were nodules (*n* = 19), pleural effusions (*n* = 6), mild airway disease (*n* = 2), mild cylindrical bronchiectasis with 2.5 mm nodules (*n* = 2), lymph nodes, (*n* = 2), latent TB (*n* = 1), possible granulomatous lesion (*n* = 1) and pulmonary embolism (*n* = 1). Cohort characteristics are described in (Table 1). Mean age was greater amongst RA-ILD patients than controls, 62.1 vs. 55.4, *p* < 0.001, males were over-represented amongst patients with ILD compared to those with RA alone 45.7% vs. 30.0%, *p* < 0.001, active or previous smoking was more frequent amongst those with RA-ILD than amongst controls, 62.4% vs. 47.3%, *p* < 0.001. Patients with RA-ILD were more likely than controls to be seropositive for each of RF and anti-CCP antibodies 88.6% vs. 72.9%, *p* < 0.001 and 88.4% vs. 68.6%, *p* < 0.001 respectively.

**Table 1:** Characteristics of the Cohort

	RA -ILD/N (%)	RA—without ILD/N (%)	*p*
Group size	210	220	
Age			
Mean Age (SD),years	62.1/200 (11.8)	55.4/218 (13.9)	< 0.001
Median Age, years (IQR)	63/200 (53–71)	56/218 (45–65)	
< 40	6/200 (3.0)	31/218 (14.2)	< 0.001
40–70	144/200 (72.0)	155/218 (71.1)	
> 70	50/200 (25.0)	32/218 (14.7)	
Gender			
Male	66/220 (30)	96/210 (45.7)	< 0.001
Female	154/220 (70)	114/210 (54.3)	
Smoking			
Pack-years Mean (SD)	17.2/119 (18.5)	7.6/158 (13.6)	< 0.001
Pack-years Median (IQR)	17.2/119 (0–30)	0/158 (0–10)	
Current/past	147/203 (62.4)	98/207 (47.3)	< 0.001
Never	56/203 (27.6)	109/207 (52.7)	
Disease-specific			
RF/negative	19/167 (11.4)	54/210 (27.1)	< 0.001
RF/weak positive	19/167 (11.4)	39/210 (18.6)	
RF/positive	129/167 (77.2)	114/210 (54.3)	
CCP/negative	18/155 (11.6)	59/188 (31.4)	< 0.001
CCP/weak positive	2/155 (1.3)	10/188 (5.3)	
CCP/positive	135/155 (87.1)	119/188 (63.3)	
Erosions	40/74 (54.1)	62/214 (29.0)	< 0.001
Biologics	79/195 (40.5)	27/204 (13.2)	< 0.001
TNF	47/195 (24.1)	83/204 (40.7)	0.001
Rituximab	40/195 (20.1)	28/205 (13.7)	0.11
Abatacept	11/195 (5.6)	5/204 (2.5)	0.10
Tocilizumab	5/195 (2.6)	11/205 (5.4)	0.15
JAK inhibitor	1/195 (0.5)	8/205 (1.9)	0.14
Methotrexate	152/205 (74.1)	188/219 (85.8)	0.002

Missing data for age were 14, Sex 1, smoking 27, DAS28 139, RF 48, CCP 93

*IQR* inter-quartile range, *RF* rheumatoid factor, *CCP* cyclic citrullinated protein, *DAS28* disease activity score. *Biologics* biologic treatment, *TNF* tumour necrosis factor, *JAK* Janus kinase inhibitor

The HRCT pattern of patients with ILD, demonstrated 119 (57%) patients had UIP pattern, 64 (30.4%) had NSIP pattern, 9 (4.3%) had a pattern consistent with organising pneumonia and 18(8.6%) were unclassified.

In univariate analysis, older age of RA onset was significantly associated with RA-ILD compared to those who were less than 40 years old [Odds ratio (OR) (95% CI) 7.39(1.96–28.05] for age > 70; and 6.59 (1.89–22.96) for ages between 40 and 70). Male gender was associated with increased risk of ILD [OR 2.19 (1.33–3.62), p < 0.002]. Smoking history (current/past) had an increased risk of ILD development [OR 3.08 (1.87–5.08), *p* <  < 0.001]. Strongly positive Rheumatoid factor and anti-CCP antibodies were also associated with increased [OR 5.89 (2.84–12.21), *p* < 0.001] and 6.76 (3.03–15.10) *p* < 0.001 respectively, (Table 2).

**Table 2:** Logistic regression models (*n* = 276) including age, gender, smoking, RF, CCP and DAS 28 > 3.2

	Univariate analysis		Multivariable analysis	
	OR (95% CI)	*p*-value	OR (95% CI)	*p*-value
Age (years)				
40–70	6.59 (1.89–22.96)	0.003	5.64 (1.52–20.91)	0.01
> 70	7.39 (1.96–28.05)	0.003	11.83 (2.81–49.88)	0.001
Gender (male)	2.19 (1.33–3.62)	0.002		
Smoking	3.08 (1.87–5.08)	< 0.001	2.29 (1.29–4.06)	0.004
RF titre				
Weak positive	1.91 (0.77–4.73)	0.16	1.09 (0.38–3.15)	0.86
Positive	5.89 (2.84–12.21)	< 0.001	3.39 (1.37–8.39)	0.008
CCP titre				
Weak positive	1.46 (0.26–8.38)	0.67	0.94 (0.13–6.68)	0.95
Positive	6.76 (3.03–15.10)	< 0.001	3.64 (1.35–9.82)	0.011
DAS 28 > 3.2	0.64 (0.36–1.16)	0.141	0.49 (0.24–1.02)	0.057

*RF* rheumatoid factor, *CCP * cyclic citrullinated protein, *DAS28* disease activity score

However, in multivariate analysis, gender lost statistical significance (supplementary data, Table 1), OR 1.60 (0.75–3.44), *p* < 0.23. Therefore, gender was not included in the final model. We included moderate disease activity DAS 28 > 3.2 in the final model because extra-articular manifestations can be present in quiescent RA.

**Fig. 1:**
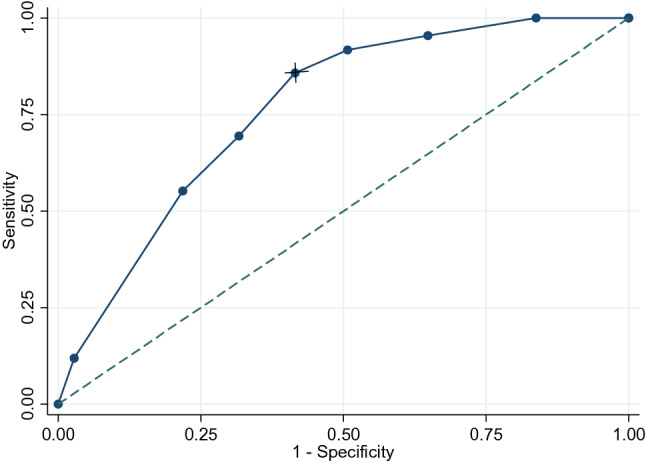
Receiver operating characteristic curve of the risk scores (the ROC area (95% CI) 0.76 (0.71–0.82). A value of 5 points (with the sign of the cross) showed a sensitivity of 86% and a specificity of 58%. The point was chosen based on the optimal sensitivity and specificity

The probability scores for RA-ILD were between 0 and 9 points that were determined by the deciles of predicted probabilities. Based on the analysis, the area under the receiver operating characteristic curve was 0.76 (CI 95% 0.71–0.82) with a sensitivity of 86% and specificity of 58% at the proposed cut-off point at 5 (Fig. 1, Tables 2, 3). The different cut-off points for smoking and DAS 28 were also evaluated in multivariable logistic analysis; similar results were observed (Supplementary tables 1, 2 and 3).

**Table 3:** The Variables used for RA-ILD probability weightage according to multivariate model

	0	1	2
Age at RA onset	< 40	40–70	> 70
Smoking	Never	Ex-smoker or current	
RF titre	Negative	Weak positive	Positive
CCP titre	Negative	Weak positive	Positive
DAS 28	DAS 28 > 3.2		

*RF* rheumatoid factor, *CCP* cyclic citrullinated protein, *DAS28* disease activity score

## Discussion

This multicentre study demonstrates that it is feasible to develop a simple scoring system to predict the probability of ILD in a patient with RA. Our four-factor score compromising smoking, age, Rheumatoid factor and anti-CCP antibodies is an easy tool to use in daily clinical practice, requiring only information that would be routinely available to the clinician. We developed the algorithm using data from a large population drawn from 20 centres over ten years. The results confirm the typical profile of an RA patient at high risk of developing ILD will be an older, smoker (past/current) with strongly positive anti-CCP antibodies and RF. At a cut-off score of 5, our model provides sensitivity of 86% and specificity of 58% for the presence of ILD. Our findings corroborate results from other studies. The relationship between tobacco exposure, anti-CCP antibody positivity and RA is well known, and smoking has been demonstrated to increase the risk of ILD [5, 10, and 27] and of other extra-articular manifestations [16].

We propose that clinicians should routinely screen for respiratory symptoms including cough, chest pain and shortness of breath along with chest auscultation for the presence of crepitations in high-risk patients. At present, there is no uniform approach to the screening of RA patients for the presence of ILD. This leads to unnecessary investigations in many and missed opportunities for early diagnosis in some. HRCT carries high specificity and sensitivity for the diagnosis of ILD and should be the gold standard for the investigation of RA patients in whom ILD is clinically suspected [2–5]. However, subjecting all RA patients to such imaging exposes the majority to unnecessary inconvenience and radiation with a low probability of identifying lung pathology in many. Hence, a simple scoring system to define which patients require screening for ILD using HRCT is much needed and is likely to optimise access to scant resources.

Early diagnosis of ILD is important in all patients as the choice of therapeutic intervention in RA is influenced by the presence of ILD. Once ILD has been diagnosed, regular pulmonary function testing can be employed to monitor progression or response to therapeutic intervention. Both vital capacity and gas transfer are highly sensitive to change but lack sufficient specificity for diagnostic purposes. Whilst the exact mechanism by which ILD occurs in patients with RA is unclear, high levels of circulating autoantibodies, particularly rheumatoid factor and anti-cyclic citrullinated peptide, combined with cigarette smoking, have been implicated as risk factors. Pérez-Dórame et al. [39] found a positive correlation between RA disease activity and HRCT using Kazerooni ground glass score in a cohort of 64 RA ILD patients, but there was no correlation in pulmonary fibrosis score. A further study found no difference between age of onset of RA with regard to DAS28 scores [10].

Paulin and colleagues [40] recently published risk scores to identify high-risk RA-ILD patients in 118 patients (of which 52 had RA-ILD). Their study variables included age, sex, presence of extra-articular manifestations, disease activity scores, antibody status, ESR and medication use. Five variables, comprising male sex, smoking, extra-articular manifestations, CDAI score > 28 and ESR > 80 were significantly associated with the presence of ILD. The AUC of the model was 0.86 (95% CI 0.79–0.92). However, their cohort was female predominant (22% male) and much smaller in sample size than ours. Furthermore, 94% of their control group had anti-CCP antibodies, which raises doubts about the patient selection and generalisability of this analysis since this number would be a lot lower in most rheumatology clinics. It is possible that the high prevalence of anti-CCP antibodies precluded a significant difference and probably caused a false negative result. This high prevalence of anti-CCP antibodies in controls is substantially greater than reported by other groups.

Kronzer et al. [41] examined several risk factors for RA-ILD including lifestyle and clinical features in a nested case–control study. This study comprised 84 RA-ILD patients, and they identified obesity, elevated CRP, poor functional status and a smoking threshold of 30 pack-years as predictors of RA-ILD. Their AUC was 0.79 and gender, age nor antibody status was significant in their model.

Narváez et al. [42] proposed screening criteria for detecting ILD in patients with RA, purely based on Delphi methodology. Their algorithm was based on similar risk factors as identified in our study. However, they recommended patients should have evidence of symptoms three months or less prior to implementing their scoring system. As stated by the authors, the usefulness of this algorithm in clinical practice will need to be validated.

Our probability score includes smoking, age, RF and anti-CCP antibodies. It has the advantage of simplicity and once validated may reduce variation in clinical assessment and aids rapid real-time decision-making. Several risk factors have previously been identified for the development of RA–ILD, but due to lack of a feasible system to accurately predict this, these patients have remained difficult to identify and risk-stratify. Q risk score for cardiovascular disease and FRAX for osteoporosis are examples of other validated scores for complications of RA that have been developed based on clinical experience and are currently being used world-wide. Similar to our model for ILD, scores are derived from simple aspects of clinical assessment. A low score allows the clinician to avoid unnecessary investigations. A high score prompts further evaluation. Patients benefit by receiving evidence-based intervention and avoiding unnecessary investigations.

There are no present guidelines for screening patients at high risk of developing ILD in RA patients. The latest American College of Rheumatology (ACR) and European League Against Rheumatism (EULAR) guidelines for the management of RA do not offer any specific advice for the detection and treatment of ILD [43, 44]. There is a need for a simple, rapid, and reliable screening tool to direct the optimal use of screening investigations, such as HRCT and pulmonary function tests. This would help facilitate the subsequent development of guidelines for monitoring and treatment of this common but life-threatening complication associated with RA.

Our four-factor probability score is an easy clinical tool for risk stratification of patients in routine clinical practice, which needs to be validated in an independent cohort. All RA patients should be considered at risk for ILD, and evaluation of lung involvement should be undertaken during routine clinical assessment because early intervention can improve quality of life. Future studies should confirm and expand the list of known risk factors, clinical, laboratory and new biomarkers through extended cohort studies. Particular focus on serum biomarkers might add further predictive value to risk prediction models influencing early diagnosis and prognosis.

Our model was derived from a large, diverse UK population of RA patients and is based entirely on clinically available data recorded in NHS healthcare records, as part of routine clinical care. The model can also be updated to take into account improvements in data quality (such as increasing numbers of patients or variables) or refined over time to reflect trends in population characteristics and changes in clinical requirements. The predictors and the weightings can be further refined in a separate larger data set with external validation.

Our model supports the Spanish algorithm [42] but takes a step further to risk-stratify patients at disease diagnosis. We have used real-term patient data to identify the risk factors and used statistical analysis to add weightage to each of the risk factor components, thereby providing a more robust corroboration of each risk factor.

Our study draws in a much larger dataset than previous RA-ILD studies to evaluate these individual risk factors. We had tighter standards for our control group criteria in comparison to previous studies, with the comparable gender and age representation in each group.

In any healthcare systems with budget constraints, our simple score based on clinical variables allows for early risk stratification and close monitoring of high-risk patients. This will help ensure high-risk individuals do not lose the opportunity for their lung disease to be identified and treated early, thus reducing their morbidity and mortality.

Given anti-fibrotics are now licenced for use in non-idiopathic progressive pulmonary fibrosis ILD, we have an opportunity for earlier diagnosis and improved outcomes for these patients.

We appreciate this study has several limitations; a number of these are due to the study design of retrospective dataset from a mixed clinical cohort. As with all observational studies, bias and incomplete data collection can affect the results. Secondly, this scoring system needs validation in an independent cohort, and it is our intention to do this. Disease duration would have been a useful addition to the data, but given the large amount of missing data within this category, it was omitted. Experience however suggests that there is no specific time point for the development of ILD, with some patients developing ILD early in disease onset.

Data regarding DMARDs, smoking status and radiological evidence of joint damage were collected at the time of evidence of ILD on HRCT. In addition, we did not have data on HAQ, body mass index and occupational history. HRCT was used for categorisation of our RA ILD cohort, but given the lack of standardisation of HRCT over the last 10 years, the data collected may have been derived from several different classifications. Nevertheless, these scans were reported by a radiologist with a specialist interest in ILD.

And finally, performance of score, sensitivity and specificity were lower in our cohort, but model calibration on a larger sample incorporating additional other markers of ILD might in the future achieve higher predictive values. Many issues have yet to be resolved, including interobserver variation in the interpretation of scans, the optimum time interval for CT follow-up and the significance of subclinical interstitial lung abnormalities. New approaches involving automated techniques and machine learning can be useful at various stages, and their significance should be investigated in the future studies.

However, in all four significantly associated variables, data ascertainment was between 90 and 100%. We have deliberately restricted our model to data collected over the most recent decade to ensure current relevance. Despite these limitations, this proof-of-concept study however strives to identify those individuals with possible RA ILD in need of further investigation. The current study only evaluated discriminative model performance.

## Conclusion

We have proposed a new model to quantify the risk of developing ILD in RA patients at point of diagnosis. Our approach uses four simple clinical variables: smoking, age, presence of Rheumatoid factor and anti-CCP antibodies to predict the risk of ILD in patients with RA.

This model requires external validation and refinement but may be applied in the clinical setting without any need for additional resource or cost implications. In the outpatient clinic setting, this simple scoring system may aid exclusion of ILD in low probability cases and facilitate further investigation in high-risk patients, ensuring scarce resources are applied optimally.

## Supplementary Information

Below is the link to the electronic supplementary material.

Supplementary file1 (DOC 88 KB)

## Data Availability

The raw data supporting the conclusions of this manuscript will be shared on reasonable request to the corresponding author.
