# Supplementary material for: Four-factor risk score for the prediction of interstitial lung disease in rheumatoid arthritis
Source: Rheumatol Int. 2023 Apr 18;43(8):1515–23. doi: 10.1007/s00296-023-05313-6 (PMC10261234; doi:10.1007/s00296-023-05313-6)
Supplement: Supplementary file 1 — Supplementary file1 (DOC 88 KB) [file 296_2023_5313_MOESM1_ESM.doc]

**Supplementary data**

Table 1: Logistic regression models (n= 195) including age, gender, smoking pack year(>20),RF, CCP and DAS 28 >3.1

|  | Univariate analysis | | Multivariable analysis | |
| --- | --- | --- | --- | --- |
|  | OR (95%CI) | p-value | OR (95%CI) | p-value |
| Age (yrs) |  |  |  |  |
| 40-70 | 13.51(1.74-104.61) | 0.013 | 9.78(1.19-80.50) | 0.034 |
| >70 | 17.88(2.14-149.52) | 0.008 | 19.75(1.98-196.77) | 0.011 |
| Gender (male) | 2.75(1.48-5.10) | 0.001 | 1.60(0.75-3.44) | 0.23 |
| Smoking park year>20 | 4.86(2.49-9.50) | <0.001 | 2.50(1.16-5.39) | 0.019 |
| RF titer |  |  |  |  |
| Weak positive | 1.77(0.56-5.56) | 0.33 | 0.85(0.21-3.49) | 0.83 |
| Positive | 6.29(2.59-15.29) | <0.001 | 3.14(0.96-10.26) | 0.058 |
| CCP titer |  |  |  |  |
| Weak positive | 3.4(0.49-23.65) | 0.22 | 3.80(0.43-33.58) | 0.23 |
| Positive | 9.46(3.20-27.97) | <0.001 | 5.35(1.33-21.54) | 0.018 |
| DAS 28>3.1 | 0.49(0.22-1.09) | 0.08 | 0.38(0.14-1.02) | 0.055 |
|  |  |  |  |  |

Figure 1: Receiver operating characteristic curve of the risk scores (the ROC area (95%CI): 0.80 (0.74-0.86). A value of 5 points (with the sign of the cross) showed a sensitivity of 88% and a specificity of 62%. The point was chosen based on the optimal sensitivity and specificity.

Table 2: Logistic regression models (n= 195) including age, gender, smoking pack year(>20),RF, CCP and DAS 28-mean

|  | Univariate analysis | | Multivariable analysis | |
| --- | --- | --- | --- | --- |
|  | OR (95%CI) | p-value | OR (95%CI) | p-value |
| Age (yrs) |  |  |  |  |
| 40-70 | 13.51(1.74-104.61) | 0.013 | 10.95(1.34-89.47) | 0.025 |
| >70 | 17.88(2.14-149.52) | 0.008 | 19.98(2.03-196.84) | 0.01 |
| Gender (male) | 2.75(1.48-5.10) | 0.001 |  |  |
| Smoking park year>20 | 4.86(2.49-9.50) | <0.001 | 2.59(1.21-5.57) | 0.014 |
| RF titer |  |  |  |  |
| Weak positive | 1.77(0.56-5.56) | 0.33 | 0.94(0.23-3.81) | 0.93 |
| Positive | 6.29(2.59-15.29) | <0.001 | 3.04(0.95-9.69) | 0.06 |
| CCP titer |  |  |  |  |
| Weak positive | 3.4(0.49-23.65) | 0.22 | 3.36(0.38-29.58) | 0.27 |
| Positive | 9.46(3.19-27.97) | <0.001 | 5.47(1.38-21.67) | 0.015 |
| DAS 28>mean | 0.82(0.46-1.44) | 0.49 | 0.56(0.27-1.13) | 0.107 |

Figure 2: Receiver operating characteristic curve of the risk scores (the ROC area (95%CI): 0.80 (0.74-0.86). A value of 5 points (with the sign of the cross) showed a sensitivity of 87% and a specificity of 62%. The point was chosen based on the optimal sensitivity and specificity.

Table 3: Logistic regression models (n= 276) including age, gender, smoking,RF, CCP and DAS 28-mean

|  | Univariate analysis | | Multivariable analysis | |
| --- | --- | --- | --- | --- |
|  | OR (95%CI) | p-value | OR (95%CI) | p-value |
| Age (yrs) |  |  |  |  |
| 40-70 | 6.59(1.89-22.96) | 0.003 | 6.15(1.67-22.68) | 0.006 |
| >70 | 7.39(1.95-28.05) | 0.003 | 10.11(2.34-43.73) | 0.002 |
| Gender (male) | 2.19(1.33-3.62) | 0.002 |  |  |
| Smoking | 3.08(1.87-5.08) | <0.001 | 2.23(1.25-3.98) | 0.007 |
| RF titer |  |  |  |  |
| Weak positive | 1.91(0.77-4.73) | 0.16 | 1.08(0.38-3.09) | 0.88 |
| Positive | 5.89(2.84-12.21) | <0.001 | 3.34(1.35-8.25) | 0.009 |
| CCP titer |  |  |  |  |
| Weak positive | 1.46(0.26-8.38) | 0.67 | 1.07(0.16-6.99) | 0.95 |
| Positive | 6.76(3.03-15.10) | <0.001 | 3.53(1.32-9.44) | 0.012 |
| DAS 28>mean | 0.77(0.48-1.23) | 0.28 | 0.58(0.33-1.02) | 0.059 |

Figure 3: Receiver operating characteristic curve of the risk scores (the ROC area (95%CI): 0.77 (0.72-0.82). A value of 6 points (with the sign of the cross) showed a sensitivity of 78% and a specificity of 61%. The point was chosen based on the optimal sensitivity and specificity.
